# Accelerometer-Measured Physical Activity and Sedentary Time Differ According to Education Level in Young Adults

**DOI:** 10.1371/journal.pone.0158902

**Published:** 2016-07-12

**Authors:** Marko T. Kantomaa, Marjaana Tikanmäki, Anna Kankaanpää, Marja Vääräsmäki, Marika Sipola-Leppänen, Ulf Ekelund, Harto Hakonen, Marjo-Riitta Järvelin, Eero Kajantie, Tuija H. Tammelin

**Affiliations:** 1 LIKES–Research Center for Sport and Health Sciences, Jyväskylä, Finland; 2 Department of Epidemiology and Biostatistics, MRC Health Protection Agency (HPE) Centre for Environment and Health, School of Public Health, Imperial College London, London, United Kingdom; 3 Chronic Disease Prevention Unit, National Institute for Health and Welfare, Helsinki and Oulu, Finland; 4 Institute of Health Sciences, University of Oulu, Oulu, Finland; 5 PEDEGO Research Unit, Medical Research Center Oulu, Oulu University Hospital and University of Oulu, Oulu, Finland; 6 Department of Children and Young People and Families, National Institute for Health and Welfare Oulu, Oulu, Finland; 7 Department of Sport Medicine, Norwegian School of Sport Sciences, Oslo, Norway; 8 Medical Research Council Epidemiology Unit, University of Cambridge, Cambridge, United Kingdom; 9 Biocenter Oulu, University of Oulu, Oulu, Finland; 10 Unit of Primary Care, Oulu University Hospital, Oulu, Finland; 11 Children’s Hospital, Helsinki University Central Hospital and University of Helsinki, Helsinki, Finland; Kent State University, UNITED STATES

## Abstract

This study examined the association of education level with objectively measured physical activity and sedentary time in young adults. Data from the Finnish ESTER study (2009–2011) (*n* = 538) was used to examine the association between educational attainment and different subcomponents of physical activity and sedentary time measured using hip-worn accelerometers (ActiGraph GT1M) for seven consecutive days. Overall physical activity, moderate-to-vigorous physical activity (MVPA), light-intensity physical activity and sedentary time were calculated separately for weekdays and weekend days. A latent profile analysis was conducted to identify the different profiles of sedentary time and the subcomponents of physical activity. The educational differences in accelerometer-measured physical activity and sedentary time varied according to the subcomponents of physical activity, and between weekdays and weekend days. A high education level was associated with high MVPA during weekdays and weekend days in both sexes, high sedentary time during weekdays in both sexes, and a low amount of light-intensity physical activity during weekdays in males and during weekdays and weekend days in females. The results indicate different challenges related to unhealthy behaviours in young adults with low and high education: low education is associated with a lack of MVPA, whereas high education is associated with a lack of light-intensity physical activity and high sedentary time especially during weekdays.

## Introduction

Contemporary data have demonstrated that, on average, adults spend approximately 60–70% of their waking hours involved in sedentary activities [1], which have been consistently associated with an increased risk of chronic disease [2–4]. It has been hypothesized that displacing sedentary time with any type of movement, including light-intensity physical activity, may have desirable health effects [5]. For example, time spent in light-intensity physical activity is beneficially associated with physical health and well-being [6], including favourable cardiometabolic biomarkers [3,7,8]. Furthermore, adherence to interventions supporting light-intensity physical activity may be higher than those interventions involving only moderate-to-vigorous physical activity as there are fewer potential barriers [5].

Education is a key social determinant of health [9,10]. Variations in physical activity and sedentary behaviour according to educational attainment are important as they may represent a causal pathway by which social inequalities lead to poor health [11]. Studies have shown that moderate-to-vigorous physical activity is more common among highly educated people than those with low levels of education [12,13]. However, there is limited evidence on the association between education and light-intensity physical activity, which is typically difficult to capture via self-reporting [14]. The association between education and sedentary behaviour may be even more complicated depending on the type of sedentary behaviour. According to recent literature, highly educated people spend more time on computers [15] but less time viewing TV [15,16]. Furthermore, the results on the association between education and sedentary time are inconsistent; some studies have reported that education level is positively associated with sedentary time [17–19], while others have not observed such an association [20–22].

The extant literature on education in association with physical activity and sedentary behaviour has relied on self-reported measures of these behaviours [12,13,17,19,23], and few studies have reported educational differences in physical activity and sedentary time measured objectively using accelerometers [24]. Objective methods (such as those that employ accelerometers) may provide more precise ways to estimate the frequency, duration, and intensity of physical activity and sedentary time than self-reporting alone [25], thus reducing the potential for measurement error and increasing the likelihood of detecting true associations between education and physical activity [26].

To develop targeted interventions to reduce excess sedentary time, it is important to understand educational differences in all dimensions of physical activity. The aim of this study was therefore to examine the association of education level with accelerometer-measured physical activity and sedentary time in young adulthood, and to evaluate the possible differences in these results between weekdays and weekend days. We hypothesized that a higher level of education is associated with high sedentary time and low overall physical activity in young adults.

## Materials and Methods

### Participants

The participants of the ESTER study comprised 1,161 young adults aged 23.3 years (SD 1.2, range 19.9–26.3) from Northern Finland. From 2009 to 2011, they participated in a clinical study that consisted of questionnaires on family structure, medical history, current health and medications, socioeconomic position and lifestyle, as well as a broad spectrum of measurements, including accelerometer-measured physical activity and sedentary time. The ESTER study originally aimed to evaluate the effects of preterm birth on health and well-being later in life and therefore has two arms with different research goals: 1) preterm birth and early life programming of adult health and disease [27], and 2) maternal pregnancy disorders and children’s health in adulthood. Among the participants, 149 were born early preterm (<34 weeks’ gestation), 248 late preterm (≥34 but <37 weeks’ gestation), 159 from pregnancies with maternal gestational diabetes, 427 from pregnancies with maternal hypertensive disorders, and 287 were randomly selected and born full term from pregnancies without any of the aforementioned pregnancy complications. The analysis in the present study included the 538 participants (224 males and 314 females) who had valid data on accelerometer-measured physical activity and sedentary time. The ESTER study conformed to the principles of the Declaration of Helsinki. The participants took part voluntarily and signed informed consent forms. The Ethics Committees at Helsinki and Uusimaa as well as the Northern Ostrobothnia Hospital District approved the research protocol.

### Physical Activity and Sedentary Time

Physical activity was measured objectively using accelerometers (ActiGraph GT1M, ActiGraph, Pensacola, Florida). Each participant wore an accelerometer on the right hip with an elastic waistband during waking hours for seven consecutive days [28]. ActiLife software version 5.4 (http://support.theactigraph.com/dl/ActiLife-software) was used to initialize the accelerometers and to download the data. Customized software was used for data reduction and analysis. The epoch length was 60 seconds, and the non-wearing time was 60 minutes. The outcome variables were overall physical activity counts per minute (cpm) (min/day), time spent on moderate-to-vigorous intensity physical activity (MVPA_1 min_; min/day, ≥1,952 cpm), continuous moderate-to-vigorous intensity physical activity lasting at least 10 minutes at a time (MVPA_10 min_; min/day, ≥1,952 cpm), which is in accordance with the current physical activity recommendation [29], time spent on light-intensity physical activity (min/day, 100–1,951 cpm) [30] and sedentary time (% of wearing time/day, <100 cpm). A one-minute interruption was allowed within a five-minute time frame. All outcome variables were computed separately for weekdays and weekend days. In addition, the variables were computed as weighted averages of daily physical activity during weekdays and weekend days (daily physical activity–[5 * average weekday physical activity + 2 * average weekend day physical activity] / 7). Participants were included in the analysis if they had valid data for at least 500 minutes per day on two weekdays and one weekend day [28]. Accelerometer-measured sedentary time was standardized with daily wearing time, which allowed for a comparison of the participants who had worn the accelerometers for different amounts of time per day.

### Education Level

Information on obtained educational qualifications was self-reported in response to the question: ‘What educational qualifications have you obtained?’ Information on educational qualifications currently taken was measured by asking: ‘What educational qualifications are you currently studying for?’ For both questions, the participants could choose multiple options from the following response alternatives: 1) comprehensive school (9 years of education), 2) vocational (11–12 years), 3) college (13–15 years), 4) upper secondary (11–12 years), 5) polytechnic (14–17 years), 6) university (16–18 years) and 7) none of the above. In Finland, higher education consists of two sectors, namely universities and polytechnics, with the latter providing vocational education on a higher level and promoting applied research. Education level was defined as the highest category of obtained qualification (or the qualification any ongoing education will lead to) and categorized according to the education level categories used by the International Standard Classification of Education [31].

### Potential Confounders

Information on employment status was self-reported in response to the following question: ‘Are you currently (mainly) 1) employed, 2) a stay-at-home mother/father, 3) a student, 4) unemployed, 5) a conscript (persons not in the labour force who are presently in military or non-military service, which is compulsory for men and voluntary for women in Finland), 6) retired or 7) other?’ Each participant’s economic situation was subjectively evaluated by asking: ‘What is your current economic situation like?’ The response alternatives were: 1) very good, 2) somewhat good, 3) average, 4) somewhat bad and 5) very bad. Due to the selection criteria of the birth cohort, participants born early or late preterm were identified after accurately determining the length of gestation, and the diagnoses of maternal gestational diabetes, hypertension or preeclampsia were retrospectively confirmed according to the prevailing criteria [32,33] and treated as potential confounders.

### Statistical Analysis

Sample characteristics were summarized descriptively, using mean and SD values for continuous data and frequencies and percentages for categorical data. The cross-sectional associations of education level with physical activity and sedentary time were examined via a linear regression analysis, stratified by sex and, further, by the days of the week (weekdays vs weekend days). The results of the regression analyses are presented with standardized regression coefficients and 95% confidence intervals (95% CI). As the original study sample was designed to study the effects of perinatal conditions on adult health and the transition to adulthood, the analyses were adjusted in the multivariable models as follows: preterm birth and maternal gestational diabetes and hypertension (Model 1), and thereafter, preterm birth, maternal gestational diabetes and hypertension, employment status and self-assessment of economic situation (Model 2). A latent profile analysis was conducted to identify the different profiles of sedentary time and physical activity of varying intensities. The classification was based only on the means, but observed variables were allowed to correlate. The number of latent classes was approximated using Akaike’s information criterion (AIC), the Bayesian information criterion (BIC) and a sample-size adjusted Bayesian information criterion. Statistical tests–the Vuong-Lo-Mendell-Rubin likelihood ratio test (VLMR), the Lo-Mendell-Rubin adjusted likelihood ratio test (LMR) and the parametric bootstrapped likelihood ratio test (BLRT)–were also applied to determine the number of clusters. Entropy was used to evaluate the classification quality. For further analysis, the participants were classified into their most likely classes. The association between educational level and the formed classification was studied using cross-tabulation and the χ^2^-test. A full information maximum likelihood estimation with robust standard errors was used under the assumption of data missing at random. The level for statistical significance was determined as *P*<0.05. The statistical analyses were conducted in 2015 using SPSS® for Windows 19.0 (IBM Corporation, Armonk, NY) [34] and the Mplus statistical package (Version 7) [35].

## Results

The sex-specific distributions of education level, overall physical activity, MVPA_1 min_, MVPA_10 min_, light-intensity physical activity and sedentary time are presented in Table 1. The weighted average of daily physical activity during weekdays and weekend days was 295 cpm for overall physical activity, 31.0 minutes for MVPA_1 min_, 10.8 minutes for MVPA_10 min_ and 274.4 minutes for light-intensity physical activity. The daily time spent sedentary was, on average, 65.4%. Fifty-seven percent of the participants had obtained or were currently pursuing a higher education (polytechnic or university) degree.

**Table 1:** Characteristics of the ESTER study participants.

Characteristics	Male (*n* = 224)		Female (*n* = 314)		All (*n* = 538)	
	*n*	%, mean (SD)	*n*	%, mean (SD)	*n*	%, mean (SD)
**Overall PA (cpm)**	224	306 (128)	314	287 (124)	538	295 (126)
**MVPA**_**1 min**_ **(min/day)**	224	34.2 (21.0)	314	28.8 (19.6)	538	31.0 (20.3)
**MVPA**_**10 min**_ **(min/day)**	224	9.6 (12.0)	314	11.6 (12.9)	538	10.8 (12.5)
**Light-intensity PA (min/day)**	224	269.1 (96.0)	314	278.2 (79.8)	538	274.4 (87.0)
**Sedentary time (% of wearing time/day)**	224	66.2 (10.7)	314	64.8 (9.0)	538	65.4 (9.8)
**Education**	224		311		534	
Primary	5	2.2	4	1.3	9	1.7
Vocational	59	26.3	69	22.2	128	24.0
Upper secondary	25	10.7	44	14.1	68	12.7
College	11	4.9	14	4.5	25	4.7
Polytechnic	68	30.4	95	30.5	163	30.5
University	56	25.0	85	27.4	141	26.4
**Employment status**	224		311		534	
Employed	78	34.8	94	30.3	172	32.0
Stay-at-homemother/father	0	N/A	26	8.4	26	4.8
Student	115	51.3	155	49.8	270	50.2
Unemployed	21	9.4	25	8.0	46	8.6
Conscript	0	N/A	2	0.6	2	0.4
Other	9	4.0	9	2.9	18	3.3
**Economic situation**	223		309		531	
Very good	4	1.8	6	1.9	10	1.9
Somewhat good	51	22.8	64	20.7	115	21.7
Average	106	47.7	129	41.8	235	44.3
Somewhat bad	55	24.8	98	31.7	153	28.8
Very bad	6	2.7	12	3.9	18	3.4
**Preterm birth**	150		203		352	
Term	76	50.7	104	51.2	180	51.1
Preterm (<34 wk GA)	24	16.0	40	19.7	64	18.2
Preterm (34 to <37 wk GA)	50	33.3	59	29.1	108	30.7
**Maternal gestational diabetes**	105		138		242	
No	78	75.0	102	73.9	180	74.4
Yes	26	25.0	36	26.1	62	25.6
**Maternal gestational hypertension**	158		217		374	
Normotensive	68	43.1	87	40.1	155	41.3
PE + superimposed PE	34	21.5	53	24.4	87	23.2
GH + CH	49	31.0	66	30.4	115	30.7
Proteinuria	7	4.4	11	5.1	18	4.8

Abbreviations: CH, chronic hypertension; cpm, counts per minute; GA, gestational age; GH, gestational hypertension; MVPA_1 min_, moderate-to-vigorous intensity PA calculated from single 1 min bouts throughout the measurement period; MVPA_10 min_, moderate-to-vigorous intensity PA calculated from bouts of PA lasting continuously for ≥10 min; PA, physical activity; PE, preeclampsia.

**Fig 1:**
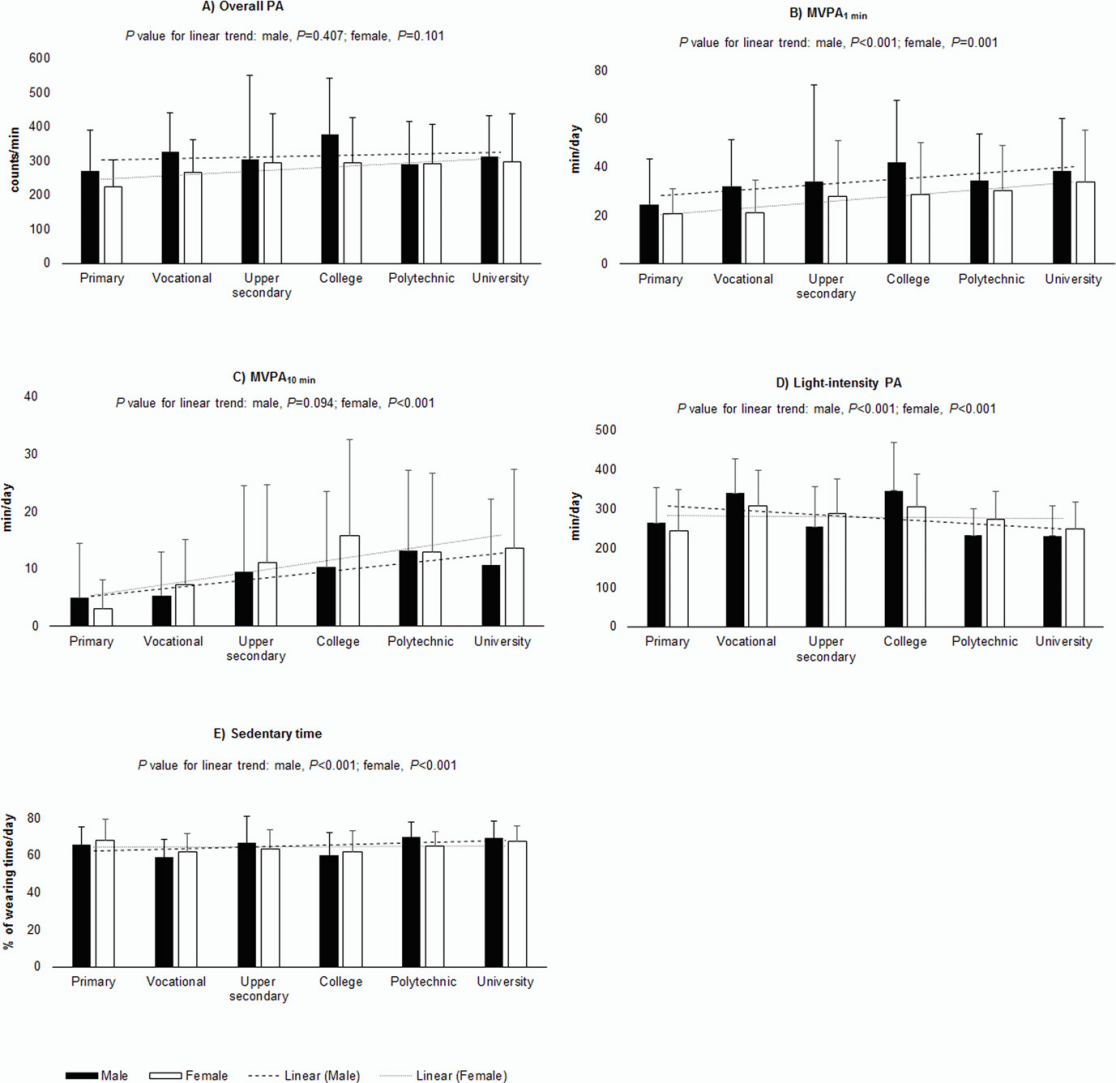
Educational differences in physical activity and sedentary time. Education level in association with overall physical activity (overall PA) (A), moderate-to-vigorous intensity physical activity calculated from single 1 min bouts throughout the measurement period (MVPA_1 min_) (B), moderate-to-vigorous intensity physical activity calculated from bouts of physical activity lasting continuously for ≥10 min (MVPA_10 min_) (C), light-intensity physical activity (light-intensity PA) (D), and sedentary time (E) in males (*n* = 224) and females (*n* = 314). The ESTER study, Finland, 2009–2011. Results of the regression analysis. Cpm, counts per minute; upper, upper secondary.

According to the unadjusted analyses, education level was not associated with accelerometer-measured overall physical activity (Fig 1A), but there was a direct educational gradient for the amount of MVPA_1 min_, MVPA_10 min_ (only females) and sedentary time, and an inverse gradient in light-intensity physical activity. The males with college education had the highest (42.1 min/day, *P*<0.001) amount of MVPA_1 min_, while the females with primary education had the lowest (20.7 min/day, *P* = 0.001) (Fig 1B). Similarly, the females with college education had the highest (15.8 min/day) amount of MVPA_10 min_ and those with primary education had the lowest (3.1 min/day, *P*<0.001) (Fig 1C), whereas the males with college education had the highest (347.0 min/day) amount of light-intensity physical activity, and those with university education had the lowest (232.0 min/day, *P*<0.001) (Fig 1D). Nevertheless, the males with polytechnic education had the highest (70.0% of wearing time/day) amount of sedentary time, and those with vocational education had the lowest (59.0% of wearing time/day, *P*<0.001) (Fig 1E).

The results of the multiple regression analysis supported the results of the unadjusted analysis, indicating that there was a direct educational gradient in the amount of MVPA_1 min_ (min/day per one step, higher education) (in *females* only: B = 2.609, *P*<0.001), MVPA_10 min_ (min/day) (*males*: B = 1.853, *P*<0.001; *females*: B = 1.725, *P*<0.001) and sedentary time (% of wearing time/day) (*males*: B = 2.277, *P*<0.001; *females*: B = 1.071, *P* = 0.004), and an inverse gradient in light-intensity physical activity (min/day) (*males*: B = –24.398, *P*<0.001; *females*: B = –14.584, *P*<0.001) after adjusting for employment status, self-assessment of economic situation, preterm birth, and maternal gestational diabetes and hypertension (Table 2). The described models explained 5–24% of the variance in physical activity and sedentary time, as indicated by the *R*^2^ values (Table 2).

**Table 2:** Regression analysis of educational level, accelerometer-based physical activity and sedentary time.

	Model 1^a^			Model 2^b^		
	B	95% CI	*R*^2^	B	95% CI	*R*^2^
**Males (*n* = 224)**						
Overall PA (cpm)	–1.555	–11.615, 8.504	0.029	–2.336	–12.701, 8.029	0.060
MVPA_1 min_ (min/day)	2.031	0.307, 3.755	0.036	1.758	–0.045, 3.560	0.051
MVPA_10 min_ (min/day)	1.895	0.944, 2.846	0.093	1.853	0.900, 2.807	0.094
Light-intensity PA (min/day)	–24.463	–31.572, –17.354	0.209	–24.398	–31.811, –16.986	0.239
Sedentary time (% of wearing time/day)	2.231	1.503, 3.117	0.165	2.277	1.423, 3.131	0.186
**Females (*n* = 314)**						
Overall PA (cpm)	7.421	–1.310, 16.151	0.040	6.930	–2.734, 16.594	0.092
MVPA_1 min_ (min/day)	2.911	1.628, 4.194	0.068	2.609	1.161, 4.057	0.104
MVPA_10 min_ (min/day)	1.651	0.885, 2.417	0.043	1.725	0.845, 2.605	0.056
Light-intensity PA (min/day)	–13.533	–19.893, –7.173	0.110	–14.584	–21.011, –8.157	0.209
Sedentary time (% of wearing time/day)	1.200	0.492, 1.909	0.087	1.071	0.339, 1.803	0.179

Abbreviations: cpm, counts per minute; MVPA_1 min_, moderate-to-vigorous intensity PA calculated from single 1 min bouts throughout the measurement period; MVPA_10 min_, moderate-to-vigorous intensity PA calculated from bouts of PA lasting continuously for ≥10 min; PA, physical activity.

^a^ Adjusted for preterm birth, and maternal gestational diabetes and hypertension.

^b^ Adjusted for preterm birth, maternal gestational diabetes and hypertension, employment status and economic situation.

When the results were stratified by the days of the week (weekday vs weekend day), an educational gradient was observed among the females during both weekdays and weekend days for MVPA_1 min_ (min/day) (weekdays: B = 2.946, *P* = 0.001; weekend days: B = 1.795, *P* = 0.031) and light-intensity physical activity (min/day) (weekdays: B = –17.301, *P*<0.001; weekend days: B = –7.587, *P* = 0.047). Among the males, an educational gradient was seen only during weekend days for MVPA_1 min_ (B = 3.255, *P* = 0.007) and only during weekdays for light-intensity physical activity (B = –31.063, *P*<0.001) (Table 3). For MVPA_10 min_ (min/day), the educational gradient remained in both the males (weekdays: B = 1.631, *P* = 0.001; weekend days: B = 2.409, *P* = 0.003) and the females (weekdays: B = 1.824, *P* = 0.001; weekend days: B = 1.490, *P* = 0.011). For sedentary time, the educational gradient was observed only during weekdays in both the males (B = 3.045% of wearing time/day, *P*<0.001) and the females (B = 1.294% of wearing time/day, *P* = 0.001) but not during weekend days. The described models explained 4–28% of the variance in physical activity and sedentary time, as indicated by the *R*^2^ values (Table 3).

**Table 3:** Regression analysis of education level and accelerometer-measured physical activity and sedentary time on weekdays and weekend days.

	Weekdays^a^			Weekend days^a^		
	B	95% CI	*R*^2^	B	95% CI	*R*^*2*^
**Males (*n* = 224)**						
Overall PA (cpm)	–2.336	–12.701, 8.029	0.060	15.052	–0.522, 30.627	0.065
MVPA_1 min_ (min/day)	1.158	–0.932, 3.248	0.039	3.255	0.897, 5.613	0.062
MVPA_10 min_ (min/day)	1.631	0.631, 2.631	0.086	2.409	0.825, 3.994	0.079
Light-intensity PA (min/day)	–31.063	–39.619, –22.507	0.284	–7.804	–16.414, 0.94	0.060
Sedentary time (% of wearing time/day)	3.045	2.069, 4.021	0.230	0.346	–0.61, 1.302	0.061
**Females (*n* = 314)**						
Overall PA (cpm)	8.095	–2.855, 19.044	0.081	4.135	–5.992, 14.262	0.095
MVPA_1 min_ (min/day)	2.946	1.264, 4.628	0.091	1.795	1.165, 3.426	0.105
MVPA_10 min_ (min/day)	1.824	0.776, 2.871	0.050	1.490	0.342, 2.637	0.057
Light-intensity PA (min/day)	–17.301	–24.229, –10.373	0.206	–7.587	–15.066, –0.108	0.120
Sedentary time (% of wearing time/day)	1.294	0.498, 2.090	0.171	0.516	–0.271, 1.302	0.109

Abbreviations: cpm, counts per minute; MVPA_1 min_, moderate-to-vigorous intensity PA calculated from single 1 min bouts throughout the measurement period; MVPA_10 min_, moderate-to-vigorous intensity PA calculated from bouts of PA lasting continuously for ≥10 min; PA, physical activity.

^a^ Adjusted for preterm birth, maternal gestational diabetes and hypertension, employment status and economic situation.

A latent profile analysis was performed for the observed variables MVPA_1 min_, MVPA_10 min_, light-intensity physical activity and sedentary time reported above. The VLMR and LMR suggested that the five-class solution was sufficient, the BIC indicated that the seven- or eight-class solution would be the best, and according to AIC, the adjusted BIC and BLRT, the class number should be even more than eight (Table 4). The seven-class solution was chosen because only small groups were extracted at the eighth and ninth steps (*n* = 4 and *n* = 7). The entropy for the seven-class solution was 0.86. Two groups were eliminated prior to further analysis because of their small sizes (*n* = 9 and *n* = 13).

**Table 4:** Classes identified through the LCA with goodness-of-fit statistics (*n* = 5538).

Number of classes	AIC	BIC	Adjusted BIC	VLMR	LMR	BLRT	Entropy
**1**	17586	17655	17601	-	-	-	-
**2**	17428	17510	17449	0.007	0.008	<0.001	0.91
**3**	17306	17409	17332	0.006	0.006	<0.001	0.94
**4**	17221	17345	17253	0.049	0.053	<0.001	0.91
**5**	17164	17310	17202	0.012	0.014	<0.001	0.93
**6**	17138	17305	17181	0.378	0.391	<0.001	0.87
**7**	17112	17301	17162	0.610	0.618	<0.001	0.86
**8**	17090	17300	17145	0.130	0.135	<0.001	0.88
**9**	17081	17312	17141	0.557	0.561	<0.001	0.87

Abbreviations: AIC, Akaike’s information criterion; BIC, Bayesian information criterion; BLRT, Parametric bootstrapped likelihood ratio test; LCA, latent class analysis; LMR, Lo-Mendell-Rubin adjusted likelihood ratio test; VLMR, Vuong-Lo-Mendell-Rubin likelihood ratio test.

**Fig 2:**
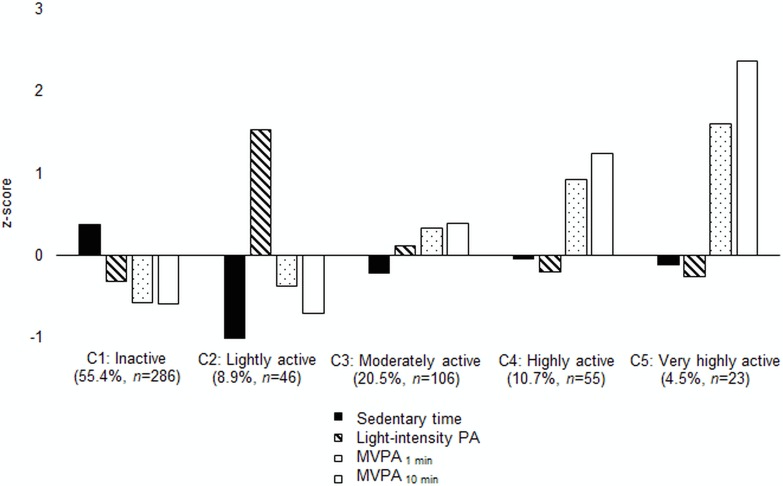
Characteristics of the classes (C1–C5) formed based on physical activity and sedentary time (*n* = 516). The mean profiles (presented in the z-score metric) of sedentary time and physical activity of different intensities. The shadings denote moderate-to-vigorous intensity physical activity calculated from single 1 min bouts throughout the measurement period (MVPA_1 min_), moderate-to-vigorous intensity physical activity calculated from bouts of physical activity lasting continuously for ≥10 min (MVPA_10 min_), light-intensity physical activity (light-intensity PA) and sedentary time.

The mean profiles of each of the five classes (C1–C5) are presented in Fig 2. The first class (CI: Inactive) was characterized by low levels of MVPA_1 min_, MVPA_10 min_ and light-intensity physical activity, and a high level of sedentary time. The participants in class 2 (C2: Lightly active) had a high level of light-intensity physical activity but low levels of MVPA_1 min_, MVPA_10 min_ and sedentary time. The third class (C3: Moderately active) was characterized by moderate levels of MVPA_1 min_, MVPA_10 min_ and light-intensity physical activity, as well as sedentary time. The participants in class 4 (C4: Highly active) had high levels of MVPA_1 min_ and MVPA_10 min_, and a moderate level of sedentary time. The fifth class (C5: Very highly active) was characterized by very high levels of MVPA_1 min_ and MVPA_10 min_, and a moderate level of sedentary time.

**Fig 3:**
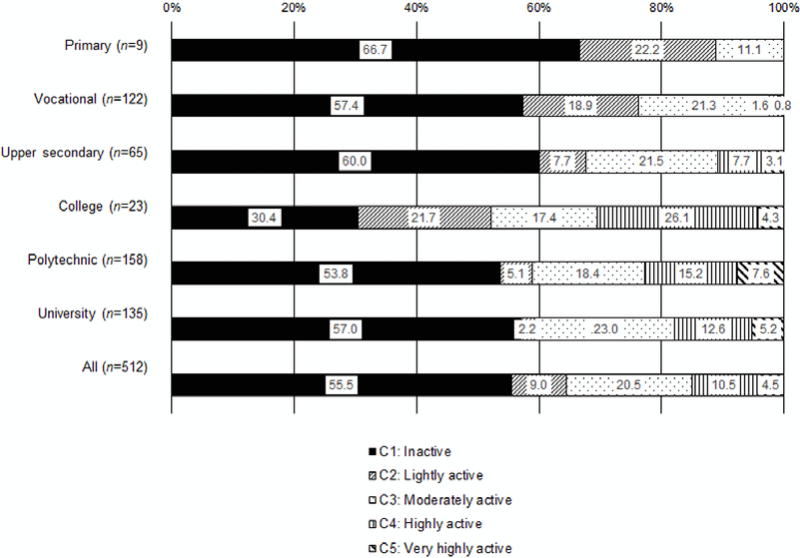
Classes formed based on physical activity and sedentary time by education level in young adults (*n* = 512). The colours denote the percentages of the class sample in class C1 (Inactive), C2 (Lightly active), C3 (Moderately active), C4 (Highly active) and C5 (Very highly active).

Education level was associated with the formed classification (*P*<0.001). The young adults with primary, vocational or upper secondary education had the highest proportion of participants in class C1 (Inactive) (Fig 3). Those with a polytechnic or university education also had a high proportion of participants in class C1, while the young adults with primary, vocational or college education had the highest proportion of participants in class C2 (Lightly active). Young adults with college, polytechnic or university education comprised the highest proportion of participants in classes C4 (Highly active) and C5 (Very highly active), whereas those with primary, vocational or upper secondary education represented the lowest proportion of participants in classes C4 and C5 (Fig 3).

## Discussion

In this study, a higher education level was associated with a higher amount of time spent on MVPA_1 min_ and MVPA_10 min_, lower light-intensity physical activity and higher sedentary time based on accelerometer-measured physical activity in young adults.

A recent Norwegian study [36] reported that adults and older people with high education levels had higher accelerometer-measured overall physical activity than those with low education levels. In the present study, education level was not associated with accelerometer-measured overall physical activity expressed as average cpm. Instead, the present results indicate educational differences in the identified subcomponents of accelerometer-measured physical activity: the levels of MVPA_1 min_, MVPA_10 min_, and sedentary time were higher, but the level of light-intensity physical activity was lower with increasing levels of education. The educational differences in MVPA_1 min_ (only females) and MVPA_10 min_ existed during both weekdays and weekend days, while the differences in light-intensity physical activity (only males) and sedentary time existed only during weekdays. Young adults with university or polytechnic education had among the highest proportion of participants engaging in high levels of MVPA_1 min_ and MVPA_10 min_, but they also had a high proportion of those classified as inactive and among the lowest proportion of those engaging in high levels of light-intensity activity. To the best of our knowledge, this is a novel finding, indicating that educational differences in accelerometer-measured physical activity and sedentary time vary according to the subcomponents of physical activity, and between weekdays and weekend days.

It is likely that different patterns of occupational physical activity largely explain the educational differences in light-intensity physical activity and sedentary time: office-based workers (typically highly educated people) spend less time in light-intensity physical activity and more time sedentary at work compared to some other occupational groups [14,37], which may not be compensated for during non-work hours or on non-work days [24,37]. Educational differences may also account for differences in health knowledge, attitudes, motivation towards physical activity and beliefs in health benefits [38], all of which may also partly explain these variations in physical activity behaviour, especially with respect to MVPA_1 min_ and MVPA_10 min_. In the present study, the patterns of accelerometer-measured physical activity and sedentary time were similar for young adults with university and polytechnic education. Notably, higher education in Finland is characterized by a strong emphasis on, and support for, health education and physical education [39].

We observed a relatively high sedentary time across all educational groups with the highest amounts among young adults with the highest education. High amounts of sedentary activities have been consistently associated with an increased risk of cardiovascular disease [40] and certain cancers [41] although it is unclear whether the associations are independent of moderate-to-vigorous intensity physical activity. Notwithstanding, interruptions in prolonged periods of sedentary time may benefit health [42]. Our result is of considerable public health and occupational health interest since it indicates that actions to reduce excessive sedentary time are needed for all educational groups. It may be beneficial to modify messages that aim to promote physical activity and reduce sedentary behaviour in specific ways for the different educational groups. Potential interventions to decrease sitting may involve breaking constant sitting with standing or light activities by, for example, restructuring the layout of offices to promote movement [43], encouraging standing rather than sitting on public transport or incorporating standing desks in the workplace [5].

Capturing different dimensions of physical activity, including sedentary behaviour and light-intensity physical activity, is the main strength of the present study. In addition, the differentiation of the results between weekdays and weekend days provides a novel point of view. Education level was defined as the highest level of both obtained education and the qualification any ongoing education would lead to, including all the main categories of the International Standard Classification of Education [31], thus potentially increasing the validity of the measure of educational attainment in this age group. However, due to the cross-sectional design of the study, conclusions regarding the causality of the observed associations could not be drawn. In addition, the originally recruited study sample (942 preterm-born individuals, 928 individuals with maternal gestational disorder during pregnancy and a random control sample of 1,050 individuals) was designed to examine the effects of perinatal conditions on adult health and the transition to adulthood, limiting the representativeness of the study sample. Nevertheless, the factors related to preterm birth and possibly confounding the association of education with physical activity and sedentary behaviour, including preterm birth and maternal gestational diabetes and hypertension, were controlled for in the final statistical models. Therefore, it is likely that our results have a general validity corresponding to similar studies [36].

The results of the present study form a basis for future research to investigate the possible causality between education level and accelerometer-measured physical activity and sedentary time. It would be useful to examine these associations in population-based study samples across different age groups and within a variety of sociocultural settings. The identification of mediating and moderating variables would be especially beneficial for physical activity interventions, which could be targeted at increasing physical activity and reducing excessive sitting within the different educational groups. In particular, information is needed across the whole intensity spectrum of these activities, as well as within the specific domains, including the different types and contexts of physical activities.

## Conclusion

A high education level among young Finnish adults was associated with high levels of accelerometer-measured moderate-to-vigorous physical activity during the whole week, but also with a lack of light physical activity and high sedentary time, mainly during weekdays. These findings may help when developing interventions aimed at increasing physical activity and reducing sedentary time among people in different educational groups.
